# Emotional processing in patients with single brain damage in the right hemisphere

**DOI:** 10.1186/s40359-022-01033-x

**Published:** 2023-01-12

**Authors:** Sonia Álvarez-Fernández, Nelson Andrade-González, Patricia Simal, Jordi A. Matias-Guiu, Carlos Gómez-Escalonilla, Roberto Rodriguez-Jimenez, Bryan J. Stiles, Guillermo Lahera

**Affiliations:** 1 Niño Jesús Children’s University Hospital, Madrid, Spain; 2 Psychiatry and Mental Health Research Group, Faculty of Medicine and Health Sciences, University of Alcalá, Alcalá de Henares, Madrid, Spain; 3 Faculty of Medicine, Alfonso X el Sabio University, Villanueva de La Cañada, Madrid, Spain; 4 Stroke Unit, Neurology Department, Hospital Clínico San Carlos, Instituto de Investigación Sanitaria San Carlos (IdISSC), Madrid, Spain; 5 Complutense University of Madrid, Madrid, Spain; 6 Instituto de Investigación Sanitaria Hospital 12 de Octubre (imas12), Madrid, Spain; 7 CIBERSAM, Madrid, Spain; 8 Department of Psychology and Neuroscience, The University of North Carolina at Chapel Hill, Chapel Hill, NC USA; 9 Faculty of Medicine and Health Sciences, University of Alcalá, Alcalá de Henares, Madrid, Spain; 10 IRyCIS, CIBERSAM, Madrid, Spain; 11 Príncipe de Asturias University Hospital, Alcalá de Henares, Madrid, Spain

**Keywords:** Ischemic stroke, Brain ictus, Right hemisphere, Social cognition, Emotion processing

## Abstract

**Background:**

The interest in the relationship between brain damage and social cognition has increased in recent years. The objectives of the present study were the following: (1) to evaluate and compare emotional facial recognition and subjective emotional experience in patients who have suffered a single ischemic stroke in the right hemisphere (RH) and in healthy people, (2) to analyze the relationship between both variables in both groups of subjects, and (3) to analyze the association between the cerebral location of the stroke and these two variables.

**Methods:**

Emotional facial recognition and the subjective emotional experience of 41 patients who had suffered a single ischemic stroke in the RH and 45 volunteers without previous cerebrovascular pathology were evaluated.

**Results:**

Brain damaged patients performed lower in facial emotional recognition and had a less intense subjective emotional response to social content stimuli compared to healthy subjects. Likewise, among patients with RH ischemic stroke, we observed negative associations between facial recognition of surprise and reactivity to unpleasant images, and positive associations between recognition of disgust and reactivity to pleasant images. Finally, patients with damage in the caudate nucleus of the RH presented a deficit in the recognition of happiness and sadness, and those with damage in the frontal lobe exhibited a deficit in the recognition of surprise, compared to those injured in other brain areas.

**Conclusions:**

Emotional facial recognition and subjective emotional experience are affected in patients who have suffered a single ischemic stroke in the RH. Professionals caring for stroke patients should improve their understanding of the general condition of affected persons and their environment, assess for risk of depression, and facilitate their adaptation to work, family, and social environments.

## Background

Ischemic stroke (IS) has been classically defined as a neurological deficit resulting from an acute episode characterized by an interruption of blood flow in the central nervous system [1]. The patient with right hemisphere (RH) IS may present with an impulsive and disinhibited behavioral style, personality and/or mood changes, irritability, alterations in sexual behavior, emotional lability, flat affect, alexithymia and low emotional facial expressivity, and anosognosia or decreased insight and difficulty in recognizing one's own limitations and impact on daily life [2–5]. Also, the RH has been shown to play a special role in the development of symptoms of mania and other bipolar syndromes in the presence of lesions located in the temporal lobe, caudate nucleus, thalamus, ventral pontine area, anterior region, and subcortical region [3, 6, 7]. It also seems to be related to the appearance of psychotic symptoms in lesions of the fronto-parietal cortex, temporo-parieto-occipital cortex and thalamus [8, 9] and with the presence of generalized anxiety disorder, apathy, alexithymia, dysphoria and sleep problems [2, 5, 10]. Depressive symptomatology, one of the most frequent outcomes after a stroke, has traditionally been related to the location of the lesion in the left hemisphere [7, 11, 12] and evidence has also been found of its relationship with the RH [2, 13]. This occurs particularly in those cases in which the predominant symptomatology is not purely emotional but cognitive and vegetative [14, 15] and in the chronic phase of stroke [16, 17].

Social cognition is also altered in stroke. Social cognition refers to the neurocognitive processes responsible for the perception, interpretation, processing, and flexible use of social information, in order to guide interpersonal behavior [18, 19]. Social cognition is responsible for the processing of social stimuli that are typically characterized by their changing nature and personal relevance, ranging from the perception of other individuals to the processing of complex social situations. The main domains of social cognition are emotion processing (EP), mentalizing, attributional bias/style, and social perception. EP is a person's ability to correctly identify the emotions of others and to manage one's own emotions. Traditionally, the RH has been considered to play a fundamental role in EP, being involved in tasks related to emotional perception, expression, and experience [20–22] especially in the presence of social interaction stimuli [23, 24]. The RH dominance hypothesis posits that this hemisphere is dominant for the perception and expression of all types of emotions, regardless of their affective valence [20, 25–32]. Some of the explanations proposed to justify this hypothesis suggest that emotion management requires functions that have been attributed to the RH, such as nonverbal, integrative, holistic, perceptual and visuospatial processing of perceived stimuli, while the left hemisphere is responsible for verbal and cognitive processing of stimuli, more distant from emotions [33].

In recent years there has been great interest in how acquired brain damage affects social cognition. The importance of emotions, empathy and social cognition in post-stroke recovery has been previously described [33–37]. However, few studies have investigated the impact of an IS on a relevant variable of social cognition (EP) and to examine the relationship between the lesion of certain brain structures and EP. Accordingly, the aims of this cross-sectional study are as follows: (1) to assess and compare emotional facial recognition and subjective emotional experience in patients who have suffered a single ischemic stroke in RH and in persons without ischemic stroke, (2) to examine the relationship between these two EP variables in both groups of subjects, and (3) to analyze the association between cerebral location of the stroke and these two variables. We hypothesized that (1) patients with a lesion after a single ischemic stroke in RH will show a lower number of hits on the facial emotion recognition task and a subjective response of lower emotional intensity than healthy subjects, (2) scores on emotional face recognition and subjective emotional response will correlate positively with each other in the two groups of participants, and (3) patients with brain damage in limbic and/or prefrontal structures will show a greater deficit in emotional face recognition and a subjective emotional response of lower intensity compared to patients with lesions in other brain areas.

## Material and methods

### Participants

We recruited 41 patients who had suffered a single ischemic stroke in the RH. The inclusion criteria for these patients were: age ≥ 18 years, ischemic lesion(s) after a single stroke, language preservation, and no history of previous cognitive impairment (as reported by the patient and/or family members). In addition, 45 volunteers with no previous cerebrovascular pathology participated in this cross-sectional study. Twenty-two of these 45 individuals lived with their own family, 14 lived in a nursing home, 6 lived alone, and 3 lived with their family of origin. In both groups, exclusion criteria included a history of head trauma, presence of moderate or severe depression, central nervous system disease, visual defect or any medical condition that could affect their cognitive performance. In the group of patients, any subject with a previous cerebrovascular pathology, diagnosed clinically or according to radiological signs, was excluded. In addition, we excluded those who had suffered lacunar strokes and those who presented signs of previous territorial cerebral infarctions. In the group of healthy subjects, we excluded persons with a history of cerebrovascular disease.

### Measures

*Oxfordshire* *Community* *Stroke* *Project* (OCSP; [38]). The OCSP is a system based on clinical criteria that distinguishes between the following categories of cerebral infarction: Total Anterior Cerebral Infarction (TACI) of frequently embolic etiology, Partial Anterior Cerebral Infarction (PACI) in which the most common etiologies are cardioembolic and atherosclerosis, and Posterior Circulation Infarction (POCI) whose most common etiology is atherosclerosis.

*Mini-Mental* *State* *Examination* (MMSE; [39]); Spanish version of Lobo et al. [40]. The MMSE measures the general cognitive status of the individual through 30 items that assess the following cognitive functions: spatial–temporal orientation, attention, concentration and memory, abstraction (calculation), language and visuospatial perception, and following basic instructions. The MMSE has shown adequate values of reliability, validity, sensitivity, and specificity in the Spanish population [40].

*Hamilton* *Depression* *Rating* *Scale* (HAM-D; Hamilton [41, 42]); Spanish version of Ramos-Brieva and Cordero-Villafáfila [43, 44]. The HAM-D measures the severity of depressive symptoms through 17 questions in which the clinician evaluates the following symptoms: depressed mood, feelings of guilt, suicidal ideation, insomnia (early, intermediate, and late types), activity level, psychomotor inhibition or agitation, psychic and somatic anxiety levels, gastrointestinal, genital or general somatic symptoms, hypochondriasis, weight loss, and level of insight. The HAM-D has shown adequate reliability, validity, and sensitivity to change in the Spanish population [43–45].

*Ekman* *60* *Faces* *Test* (EK-60F; Ekman and Friesen [46]). Spanish version of Molinero et al. [47]. The EK-60F measures the ability to recognize emotions through 60 black and white photographs of 8 × 10 cm each. These photographs show the faces of 10 actors (6 women and 4 men) expressing 6 basic emotions: happiness, sadness, disgust, fear, surprise, and anger. After randomly presenting the photographs, the participant must choose which emotion is expressed in each of them; the response options are the 6 emotions mentioned. The total score for this test is 60 points, based on the number of correct answers. The EK-60F has demonstrated adequate reliability and validity values in clinical and community samples in different countries; in Spain, normative data have been published in adolescent population [47].

*International* *Affective* *Picture* *System* (IAPS; Lang et al. [48, 49]); Spanish version of Moltó et al. [50, 51]. The IAPS has 1196 color photographs with content ranging from everyday scenes and objects to rare and potentially disturbing images. In the present study, we selected 54 photographs from the IAPS (following the procedure of Bradley and Lang [52]; Hempel et al. [53]; Sánchez-Navarro et al. [54]) which were divided, according to Spanish normative data, into 3 categories: 18 pleasant, 18 neutral and 18 unpleasant photographs. To evaluate the subjective emotional experience, the participant must rate each picture on a scale ranging from 1 (very unpleasant) to 9 (very pleasant). In addition, we divided these same photographs into two other categories: social (two or more people in a social interaction situation) and non-social. The IAPS has been used and validated in different countries; in Spain, normative scores have been obtained [50, 51, 55].

*Modified* *Rankin* *Scale* (mRS; [56]). In the mRS, clinicians globally assess the degree of physical disability in patients after stroke through seven levels of severity, from the "mild" category (0 points) to the "death" category (6 points). In this study, we grouped the levels of disability into 3 categories: mild (0–2 points), moderate (3 points), and severe (4–5 points).

*National* *Institute* *of* *Health* *Stroke* *Scale* (NIHSS; [57]); Spanish version of Montaner and Alvarez-Sabín [58]. NIHSS is a systematic assessment tool that provides a quantitative measure of stroke-related neurologic deficit through 11 items that explore: cortical functions, motor function, upper cranial nerves, language, sensitivity, and coordination. In the present study, we grouped severity levels into 4 categories: mild (0–3 points), moderate (4–15 points), severe (16–20 points), and very severe (> 20 points) deficits. The NIHSS has shown good psychometric properties in Spanish population [58].

### Procedure

Patients were recruited from the Cerebrovascular Pathology Unit of the Hospital Clínico San Carlos in Madrid, Spain using non-probability accidental sampling (convenience sample). Eligible patients signed an informed consent form and the study was approved by the Ethical Committee for Clinical Research of the aforementioned hospital. Healthy subjects were matched for age, sex, and education level with the patient sample. In both groups, we conducted an interview assessing sociodemographic variables and the following clinical variables: alcohol and tobacco consumption, beta-blocker and beta-agonist medication intake, and presence of past and current mental disorder. In addition, we assessed the cognitive status with the MMSE (only as a screening test), and the presence of depression with the HAM-D.

In the group of patients, the location of the brain lesion was determined from their clinical history and at least one computed tomography (CT) and/or magnetic resonance imaging (MRI) according to the following classification: frontal, parietal, temporal, occipital, lenticular, caudate, thalamus, insular cortex or limbic system. Following the acute ischemic stroke care protocols implemented in our hospital, we performed a baseline brain CT scan and CT-angiography to assess the supra-aortic trunks and the circle of Willis from the aortic arch to the cranial vertex. The contrast medium used was Optiray Ultraject^®^ 300 mg/mL. MRI was acquired using a 1.5 T scanner (Signa HDxt, GE Healthcare, Milwaukee, USA). The location of the lesion of 14 patients was determined by CT and MRI; the lesion of 26 patients was determined only by CT, and the lesion of 1 patient was determined only by MRI. We used these same imaging tests to determine the territory of the cerebral artery affected in the stroke (anterior, middle, or posterior cerebral artery). The type of cerebral infarction was classified by OCSP criteria. We measured the degree of physical disability after stroke with the mRS and the severity of the cognitive deficit with the NIHSS.

Emotional facial recognition and subjective emotional experience were then assessed. The mean number of days between the time of the stroke of 35 patients and the time of this evaluation was 318.9 days (*SD* = 147.4). For assessment of emotional facial recognition and subjective emotional experience, all participants sat approximately 50 cm from a computer monitor (1080 × 720 ppi resolution) projecting the EK-60F and IAPS photographs, at an angle of about 9° × 7° from the participants view. The photographs were presented with similar characteristics in terms of luminance and contrast. The EK-60F photographs were presented randomly. In the case of the IAPS, we created three PowerPoint presentations, each containing the same photographs (54 in total) but arranged differently to eliminate the potential effect of presentation order. Each of these three series of photographs began with a different image. The series were randomly assigned to subjects. Each subject was presented with only one of these series of photographs. We asked subjects to rate the photographs according to their affective valence (pleasant, neutral, or unpleasant).

### Statistical analysis

We performed data analysis with the IBM SPSS Statistics version 24 program. In the IAPS, we calculated the arithmetic mean of the values assigned to the photographs, rated by each subject in both groups as unpleasant, pleasant and neutral, and the mean of all ratings in this dimension (global subjective emotional reactivity). Next, each participant's ratings (affective valence) of the same IAPS photos were taken into account, ordered this time according to their social (e.g., social scenes depicting people) and non-social (e.g., objects depicted) content. For each participant in the two groups, we calculated the arithmetic mean of the values assigned to the social and non-social photographs. Comparison between the group of stroke patients and the group of healthy subjects on sociodemographic and clinical variables was performed with *χ*^2^ or Student's *t-*tests. To compare emotional facial recognition and subjective emotional experience between the two groups and within each group, a two-factor analysis of variance (ANOVA) was performed. Post hoc analyses were performed using Bonferroni and Tukey corrections. Given the brain-level differences related to sex, as well as the influence of this variable on neural plastic capacity and aging [59–63], it was decided to include this variable as a factor in the ANOVAs. To examine the relationship between emotional facial recognition and subjective emotional experience in the two groups of subjects separately, we used Pearson correlation coefficients. Finally, to examine the brain localization of emotional facial recognition and subjective emotional experience in the patient group, we used the nonparametric Mann–Whitney *U* test comparing the presence versus absence of lesion for each brain region. The significance level in all hypothesis contrast tests was 0.05.

## Results

Table 1 shows the sociodemographic characteristics of the participants in both samples. Table 2 includes results about location of the lesions in the group of patients. As shown, multiple regions which overlap with each other were affected although they were caused by only one stroke episode. At the end of this Results section, we examine the association between patients' lesion location and the emotional facial recognition and the subjective emotional experience. In the group of healthy subjects there were more people living in a residence, and in the group of patients there were more people living with their own family. As for the clinical variables, there were only significant differences between the two groups in tobacco use since healthy subjects smoked significantly more than the patients (*χ*^2^ = 5.87; *p* = 0.02).

**Table 1:** Sociodemographic characteristics of the participants

	Patients (*n* = 41)	Healthy subjects (*n* = 45)		
	Mean (SD) or N (%)	Mean (SD) or N (%)	*t*/*χ*^2^	*p*
Age	68.5 (12.2)	66.3 (17.8)	0.69	0.48
Males	21.0 (51.1)	23 (51.1)	0.00	0.99
Highest education level			0.45	0.79
No studies or elementary	23.0 (56.1)	23.0 (51.1)		
High school	12.0 (29.3)	13.0 (28.9)		
University	6.0 (14.6)	9.0 (20.0)		
Civil status			6.48	0.16
Single	1.0 (2.4)	8.0 (17.8)		
Married	29.0 (70.7)	26.0 (57.8)		
Widower	9.0 (22.0)	10.0 (22.2)		
Separated	1.0 (2.4)	1.0 (2.2)		
Divorced	1.0 (2.4)	0.0 (0.0)		
Living arrangement			17.42	0.00
Alone	6.0 (14.6)	6.0 (13.3)		
Family of origin	1.0 (2.4)	3.0 (6.7)		
Own family	34.0 (82.9)	22.0 (48.9)		
Residency	0.0 (0)	14.0 (31.1)

**Table 2:** Brain location of the lesion caused by a single ischemic stroke in the right hemisphere

Location of the lesion	Patients	
	*N*	%
Frontal lobe	20	50
Parietal lobe	25	62.5
Temporal lobe	22	55
Occipital lobe	7	17.9
Lenticular fasciculus	13	33.3
Caudate nucleus	6	15.4
Insular cortex	13	33.3
Thalamus	3	7.7
Limbic system and basal ganglia	22	56.4

### Emotional facial recognition and subjective emotional experience in patients and healthy subjects

Table 3 shows the mean scores of patients and healthy subjects on the EK-60F and on the IAPS. In terms of facial recognition, healthy subjects exhibited a higher number of hits on the total EK-60F (*F* = 6.90; *p* = 0.01) and, specifically, on pictures expressing happiness (*F* = 6.42; *p* = 0.01), sadness (*F* = 5.91; *p* = 0.01), and anger (*F* = 4.31; *p* = 0.04) than patients who had suffered a stroke. An intra-group comparison revealed that patients (*F* = 31.34; *p* = 0.00) and healthy subjects (*F* = 37.37; *p* = 0.00) had a higher number of hits in the recognition of the facial expression of happiness than in the recognition of all other emotions.

**Table 3:** Mean scores of patients and healthy subjects on the EK-60F and the IAPS

EK-60F	Patients	Healthy subjects		
Type of facial expression	Mean (SD)	Mean (SD)	*F*	*p*
Total score	35.3 (11.0)	41.1 (9.0)	6.90	0.01
Happiness	0.8 (0.2)	0.9 (0.9)	6.42	0.01
Fear	0.4 (0.2)	0.4 (0.2)	0.39	0.53
Surprise	0.7 (0.2)	0.8 (0.2)	3.06	0.08
Sadness	0.5 (0.2)	0.6 (0.2)	5.91	0.01
Disgust	0.6 (0.2)	0.7 (0.2)	3.36	0.07
Anger	0.4 (0.2)	0.5 (0.2)	4.31	0.04

IAPS	Patients	Healthy subjects		
Type of photographs	Mean (SD)	Mean (SD)	*F*	*p*
Total score	4.5 (0.4)	4.6 (0.4)	2.19	0.14
Pleasant	6.1 (1.2)	6.6 (1.1)	2.96	0.08
Unpleasant	2.1 (0.7)	2.2 (0.6)	0.06	0.80
Neutral	5.2 (0.5)	5.1 (0.3)	0.83	0.36
Social photographs	4.4 (0.9)	4.9 (1.0)	5.55	0.02
Non-social photographs	4.5 (0.4)	4.5 (0.2)	0.31	0.57

Regarding the subjective emotional experience, although we found no significant differences between patients and healthy subjects regarding the total mean of evaluations of all IAPS images, men in both groups rated the set of images and, in addition, the pleasant images more positively than women (*F* = 9.93; *p* = 0.00 and *F* = 9.79; *p* = 0.00 respectively). Likewise, healthy persons rated social images more positively than patients (*F* = 5.55; *p* = 0.02) while males in both groups rated them more positively than females (*F* = 16.03; *p* = 0.00). An intra-group comparison revealed that patients (*F* = 196.01; *p* = 0.00) and healthy subjects (*F* = 369.94; *p* = 0.00) rated pleasant images more positively than neutral and unpleasant ones and, furthermore, healthy males (*F* = 9.21; *p* = 0.00) gave more positive ratings to social images than to nonsocial ones.

Within the group of patients, individuals with a severe level of disability (*χ*^2^ = 6.08; *p* = 0.04) and individuals with a very severe cognitive deficit after stroke (*χ*^2^ = 6.05; *p* = 0.04) rated unpleasant IAPS images closer to neutral than other patients, suggesting a subjective emotional reaction of lower intensity. Similar results were found for the severity of the neurological deficit in the case of non-social imagery (*χ*^2^ = 8.86; *p* = 0.01). In addition, negative correlations were found between the levels of disability and severity of the neurological deficit (at discharge) and the total facial recognition score (*r* = − 0.37; *p* = 0.01 and *r* = − 0.04; *p* = 0.00, respectively). Finally, individuals with a mild level of physical disability according to the mRS (*χ*^2^ = 6.70; *p* = 0.03) and with cognitive deficit of moderate severity at discharge according to the NIHSS (*χ*^2^ = 5.79; *p* = 0.01) obtained a higher number of hits in the recognition of happiness than the rest of the patients.

### Relationship between emotional facial recognition and subjective emotional experience in patients and healthy subjects

In the patient group, we found no significant relationship between total scores on emotional facial recognition and subjective emotional experience. However, total scores on emotional recognition tended to be associated with pleasant picture appraisals (*r* = 0.30, *p* = 0.056) while we found significant relationships between surprise recognition scores and unpleasant picture appraisals (*r* =  − 0.37; *p* = 0.01), and between disgust recognition scores and pleasant picture appraisals (*r* = 0.36; *p* = 0.02). In the group of healthy subjects, we did not find a significant relationship between total scores on emotional facial recognition and subjective emotional experience. However, we found significant relationships between total scores on emotional recognition and evaluations of unpleasant and neutral images (*r* = 0.30; *p* = 0.04 and *r* =  − 0.39; *p* = 0.01 respectively). Similarly, among healthy subjects, we found significant relationships between disgust and anger recognition scores with unpleasant image appraisals (*r* = 0.39; *p* = 0.00 and *r* = 0.29; *p* = 0.05 respectively) and between fear and surprise recognition scores with neutral image appraisals (*r* =  − 0.35; *p* = 0.02 and *r* =  − 0.42; *p* = 0.00, respectively).

### Brain localization of stroke and emotional facial recognition and subjective emotional experience

**Fig. 1:**
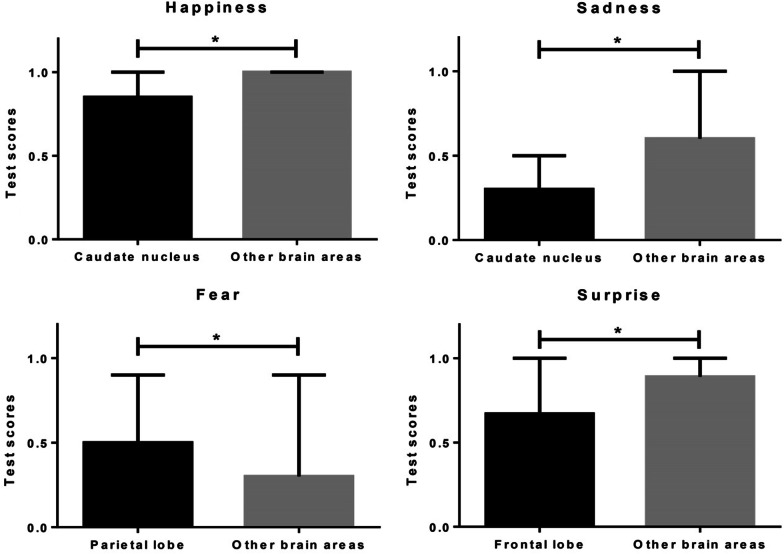
Median of hits in facial recognition of happiness, sadness, fear, and surprise according to the location of the lesion following stroke in the right hemisphere; * = *p <* 0.05

Regarding emotional facial recognition (see Fig. 1), patients with damage in the caudate nucleus presented a lower number of hits in facial recognition of happiness and sadness than those lesioned in other brain areas (*U* = 42.5; *p* = 0.01 and *U* = 34.5; *p* = 0.01 respectively). In addition, those with parietal lobe damage exhibited more hits in fear recognition than those without damage in this area (*U* = 110.5; *p* = 0.03). Finally, individuals with frontal lobe damage were less successful in recognizing surprise than those with lesions in other areas (*U* = 123.5; *p* = 0.03). As for the subjective emotional experience of the patients, we found no significant differences in the evaluation of the images when comparing the different locations of the brain lesion.

## Discussion

In this study, we examined and compared two EP variables, emotional facial recognition and subjective emotional experience, in patients who had suffered a single ischemic stroke in the RH and in subjects without neurological lesions. Both groups were matched in terms of age, sex, presence of psychopathology, cognitive level, and educational level. In addition, we analyzed the relationship between these two EP variables in both groups of subjects and the association between the cerebral location of the stroke and these two variables.

### Emotional facial recognition and subjective emotional experience in patients and healthy subjects

First, we found that healthy subjects performed better in the test of emotional facial recognition than the RH stroke patients, suggesting an alteration of this ability in people with this neurological deficit. These results are consistent with the RH dominance hypothesis, which posits a specialization of this hemisphere for the different abilities related to emotion processing, including the perception of positive and negative emotions, especially in the presence of facial and prosodic stimuli and, therefore, with a nonverbal component [28–30, 33, 64]. On the other hand, subjects in both groups recognized facial expression of happiness to a greater extent than all other emotions, a response pattern similar to that found in other studies [47, 65, 66].

Second, the absence of significant differences between patients and healthy subjects regarding their subjective emotional response to the presentation of imagery may have been due to the relatively good preservation of the stroke-affected group, as patients had preserved language and no history of prior cognitive impairment. However, the fact that males in both groups rated the set of images more pleasantly than females is consistent with some studies that have found a higher intensity of subjective response in females to stimuli with negative affective valence and a higher emotional intensity in males to stimuli with positive affective valence [67–72]. On the other hand, healthy subjects rated images of social content significantly more positively than patients. This finding is consistent with prior studies showing a relationship between RH and the processing of social information at the attentional and emotional levels [23, 24]. Likewise, the fact that males in both groups presented a more positive subjective emotional response to social content images than females may be explained by the presence of a greater number of photographs with positive valence and erotic content among the selected social content images.

### Relationship between emotional facial recognition and subjective emotional experience in patients and healthy subjects

A detailed analysis of the relationship between the different dimensions of these variables showed results of interest. Specifically, we observed significant relationships (1) in the patient group, between scores on recognition of surprise and disgust, and ratings of unpleasant and pleasant images respectively, (2) in the group of healthy subjects, between total scores on emotional facial recognition and ratings of unpleasant and neutral images, and (3) in the group of healthy subjects, between recognition of disgust and anger and ratings of unpleasant images, and between recognition of fear and surprise and ratings of neutral images. Although these findings are consistent with studies suggesting a relationship between emotional facial recognition and emotional experience (e.g., Buchanan, et al. [73]; Calder et al. [74]) these results should be interpreted with caution, given that no significant relationship was found between total scores on emotional facial recognition and subjective emotional experience in the two groups of participants, and the relatively small sample size of both groups.

### Brain localization of stroke and emotional facial recognition and subjective emotional experience

We found that patients' brain lesions located in different areas were related to facial recognition of different emotions. These results are consistent with other studies that relate emotional perception and identification to subcortical areas (such as the caudate nucleus and surrounding areas) and to temporal and frontal cortical areas [75–87]. On the one hand, this emphasizes the role of both cortical and subcortical regions of the RH in facial emotion recognition. On the other hand, our findings provide preliminary evidence about the differential role of brain regions in specific emotions in stroke patients, as has been suggested in healthy subjects using functional MRI experiments [88]. Future studies that incorporate a more detailed characterization of stroke brain injury in the RH in a larger sample of patients with more extensive lesions are needed to clarify the relationship between stroke location and subjective emotional experience. As well, other behavioral measures related to emotional processing, such as time reaction to stimuli, should be included in future research.

A deficit in facial emotion recognition in individuals with a single RH stroke has important clinical implications. This deficit implies difficulties in social interactions, isolation, problems in conflict resolution, frustration in interpersonal relationships, feelings of discomfort, and social disconnection [33, 89, 90]. Moreover, the fact that patients showed a lower subjective emotional response to social stimuli than healthy subjects suggests an attenuated emotional response to social situations that may impact on quality of life. Since depression is the most common neuropsychiatric sequelae after stroke [6, 7], it is possible that these social cognitive impairments may heighten the risk of experiencing depression especially after a severe ischemic stroke event. Considering the findings of this cross-sectional study, professionals caring for stroke patients should improve their understanding of the general condition of the persons and their environment, assess for risk of depression, and facilitate their adaptation to work, family and social environments.

The present investigation has the following limitations. First, a significant percentage of healthy individuals lived in a nursing home, which could imply a lower degree of functionality with respect to the group of stroke patients. Second, the sample size was small when intra-group comparisons were performed. Third, in the group of patients, we assessed their cognitive status with the MMSE (only as a screening test), a tool that is less sensitive than the Montreal Cognitive Assessment (MoCA) in screening for cognitive impairment after acute stroke [91]. Fourth, the lesion of 26 patients was determined only by CT, a less informative tool than MRI. Fifth, the number of photographs in the social category was lower than the number of non-social photographs and they had different proportions of images with positive and negative emotional valence. Sixth, neuroimaging assessment was based on visual reads and qualitative evaluation of several prespecified brain regions. Finally, it is possible that between the imaging test performed on individuals with stroke and the assessment phase of this cross-sectional study, clinically silent ischemic strokes may have occurred, producing subtle neuropsychological alterations.

## Conclusions

Patients with brain damage secondary to RH ischemic stroke display lower performance in emotional facial recognition compared to healthy subjects without brain damage. In addition, these patients present a subjective emotional response of lower intensity to stimuli with social content compared to healthy subjects. Likewise, males present a more positive subjective emotional response than females, regardless of the existence of brain damage in the RH. We observed that patients with brain damage due to RH ischemic stroke negatively associate facial recognition of surprise with unpleasant images and positively associate facial recognition of disgust with pleasant images. Finally, patients with damage to the caudate nucleus of the RH present a deficit in the recognition of happiness and sadness and those with damage to the frontal lobe of the same hemisphere present a deficit in the recognition of surprise. Future research comparing EP in RH and left hemisphere stroke, and/or combining multiple neuroimaging techniques (e.g., structural, diffusion-tensor imaging, functional MRI) in patients who have suffered a single ischemic stroke in the RH is necessary to further understand the neural underpinnings of EP in stroke patients.

## Data Availability

The datasets used and/or analysed during the current study are available from the corresponding author on reasonable request.

## Abbreviations

IS: Ischemic stroke
RH: Right hemisphere
EP: Emotion processing
OCSP: Oxfordshire Community Stroke Project
TACI: Total Anterior Cerebral Infarction
PACI: Partial Anterior Cerebral Infarction
POCI: Posterior Circulation Infarction
MMSE: Mini-Mental State Examination
HAM-D: Hamilton Depression Rating Scale
EK-60F: Ekman 60 Faces test
IAPS: International Affective Picture System
mRS: Modified Rankin Scale
NIHSS: National Institute of Health Stroke Scale
CT: Computed tomography
MRI: Magnetic resonance imaging
ANOVA: Analysis of variance
MoCA: Montreal Cognitive Assessment
